# Amyloid-Precursor-Protein-Lowering Small Molecules for Disease Modifying Therapy of Alzheimer's Disease

**DOI:** 10.1371/journal.pone.0082255

**Published:** 2013-12-18

**Authors:** Sina Cathérine Rosenkranz, Markus Geissen, Kristina Härter, Beata Szalay, Isidro Ferrer, Jana Vogel, Stephen Smith, Markus Glatzel

**Affiliations:** 1 Institute of Neuropathology, University Medical Center Hamburg-Eppendorf, Germany; 2 Department of Neurology, University Medical Center Hamburg-Eppendorf, Germany; 3 Department of Vascular medicine, University Medical Center Hamburg-Eppendorf, Germany; 4 Institute of Neuropathology, Hospital Universitari de Bellvitge, Hospitalet de Llobregat, Barcelona, Spain; 5 European Screening Port GmbH, Hamburg, Germany; National Center for Geriatrics and Gerontology, Japan

## Abstract

Alzheimer's disease (AD) is the most common form of dementia in the elderly with progressive cognitive decline and memory loss. According to the amyloid-hypothesis, AD is caused by generation and subsequent cerebral deposition of β-amyloid (Aβ). Aβ is generated through sequential cleavage of the transmembrane Amyloid-Precursor-Protein (APP) by two endoproteinases termed beta- and gamma-secretase. Increased APP-expression caused by APP gene dosage effects is a risk factor for the development of AD. Here we carried out a large scale screen for novel compounds aimed at decreasing APP-expression. For this we developed a screening system employing a cell culture model of AD. A total of 10,000 substances selected for their ability of drug-likeness and chemical diversity were tested for their potential to decrease APP-expression resulting in reduced Aβ-levels. Positive compounds were further evaluated for their effect at lower concentrations, absence of cytotoxicity and specificity. The six most promising compounds were characterized and structure function relationships were established. The novel compounds presented here provide valuable information for the development of causal therapies for AD.

## Introduction

Alzheimer's disease (AD) is the most common neurodegenerative disease [1]. Symptoms include cognitive dysfunction, behavioral disturbances and difficulties with performing activities of daily living [2]. Probable AD is diagnosed by presence of characteristic neurological and neuropsychological features and auxiliary tests such as neuroimaging and cerebrospinal fluid analyses, but the definite diagnosis of AD can only be made postmortem. The neuropathological hallmarks of the disease include brain atrophy, the presence of intracellular neurofibrillary tangles consisting of hyperphosphorylated tau-protein and extracellular deposition of β-amyloid (Aβ) [3].

Aβ is generated by post-translational processing of the Amyloid-Precursor-Protein (APP), a transmembrane protein, implicated in synapse formation [4] and trophic support [5]. There are two cleavage pathways of APP, a nonamyloidogenic and an amyloidogenic pathway [6]. Nonamyloidogenic cleavage of APP, by α-secretase and the γ-secretase complex releases an APP intracellular domain (AICD), the 23–25 amino-acid long p3 fragment and soluble APP (sαAPP). Amyloidogenic cleavage of APP by β-site APP cleaving enzyme 1 (BACE1) and the γ-secretase complex generates Aβ-alloforms ranging from 38 to 43 (Aβ_38_–Aβ_43_) amino acid length [7]. The main alloforms of Aβ in amyloid deposits are 40 (Aβ_40_) and 42 (Aβ_42_) acids long. Besides this, a soluble N-terminal APP fragment (sAPPβ) and AICD is produced. According to the amyloid hypothesis, generation and tissue deposition of Aβ is causal for neurodegeneration with Aβ_42_ aggregating readily and possessing high neurotoxicity [8]. Current hypothesis to explain Aβ-induced neurodegeneration include direct toxicity via the mitochondrial apoptotic pathway [9] or through activation of caspases [10],[11] and receptor mediated toxicity involving the N-methyl-D-aspartate receptor (NMDAR) [12].

The majority of AD cases are sporadic and show an association to the apolipoprotein E (APOE) ε4 allele as a genetic risk factor [13],[14]. Familial AD (FAD) is an autosomal dominant disorder with early disease onset. FAD is associated with mutations in presenilin-1 (PS1), presenilin-2 (PS2) or APP genes [15],[16],[17]. Recently copy number variations of APP have been shown to be causative for AD [18],[19]. The importance of APP gene dosage effects for the development of AD has been studied extensively in trisomy 21 patients where triplication of chromosome 21 including the APP-gene locus invariably leads to early-onset AD [20],[21],[22],[23].

Presently, there are no validated and licensed Aβ-lowering therapeutics. Efforts to develop drugs which specifically target BACE1 or the γ-secretase complex are complicated due to the pleotropic effects of these proteases leading to dramatic side effects [24],[25],[26],[27].

Moderate decrease of APP expression seems to be an attractive target for AD therapy. Therefore we screened for APP-lowering compounds using a newly developed, cell-based screening method. Of 10,000 high diversity, quality drug-like and lead-like compounds, we identified five which were non-cytotoxic, were effective at lower concentrations and lead to a selective reduction of APP and its cleavage product Aβ. Our data opens a new therapeutic approach by targeting APP and may lead to development of novel drugs to treat AD.

## Materials and Methods

### Cell culture

Human embryonic kidney 293 (HEK) cells [28] were grown in Dulbecco's modified Eagle's medium high glucose with L-glutamine, supplemented with 10% fetal bovine serum, 1% penicillin/streptomycin (PAA Laboratories, Paching, Austria) in a 5% CO_2_ incubator. The APPsw cells (gift from C.Haass), are stably transfected HEK cells overexpressing APP with a double mutation at codons 670/671 and 5–8 fold increase in Aβ production when compared to wild type HEK-cells [29]. They were grown as described above with supplemental 1% Gentamycin (G418; PAA Laboratories). N2a cells (mouse neuroblastoma cell line) were grown as described above without G418. N2a cells were stably transfected with APPsw using the FUGENE®HD transfection kit, cells were grown with G418. APPsw or N2asw cells were plated on 96-well plates (100 µl/well = 30.000 cells/well). After 1 day, compounds (one compound/well) were added to media, untreated controls received solvent only. Initial concentrations were 100 µM (in 1% Dimethyl sulfoxide (DMSO)) [30]. To check effects at lower concentrations, compounds were used at concentrations of 100 µM, 50 µM, 10 µM and 1 µM. Supernatants (for Dot blot, Western blot and ELISA) or cells (for Western blot and quantitative reverse transcription PCR) were collected after 3 days of incubation.

### Immunofluorescence

To assess transfection-efficiency, cells were grown on cover slips, fixed in acetone (20 min at −20°C), rinsed with phosphate-buffered saline, pH 7.4 (PBS, PAA Laboratories) and blocked with 5% donkey serum (Dianova, Hamburg, Germany). As primary antibody, anti-APP/Aβ antibody 6E10 recognizing the first 16 amino acids of Aβ was used (1∶200, Covance, Princeton, USA). After washing with PBS the secondary antibody (1∶500, AlexaFluor 488-labled donkey anti-mouse, Invitrogen, Carlsbad, USA) and 4′6-Diamidino-2′-phenylindole (DAPI, 1∶200 Roche, Munich, Germany) were added, coverslips were embedded in Fluoromont G (Biozol, Munich, Germany) and analyzed by fluorescence microscopy (Leica Laser Scanner Confocal Microscope TCS SP2 (Leica, Wetzlar, Germany)).

### Compound library

The compound library DIVERSet™ (Chembridge, San Diego, USA) consists of 10,000 substances supplied in 125 96-well-plates with 80 compounds each at a concentration of 2 µmol, solved in 200 µl DMSO. Due to their structural characteristics all substances are predicted to fulfil the Lipinski's rule of five for drug-likeness. For detailed information (including structural data) see: www.chembridge.com.

### Dot blot analysis

Dot blots to assess soluble α-APP were performed as published [31]. Briefly, polyvinylidene difluoride (PVDF) membrane (Biorad, Munich, Germany) was rinsed in 10 ml of methanol for 3 sec, equilibrated in PBS for 1 min, and placed in a dot blot apparatus (TE70 ECL Semi-Dry Transfer Unit, Amersham Biosciences, Freiburg, Germany). The supernatant of each well was spotted with an Eppendorf Multipipette onto the PVDF membrane through the sample wells of the Dot blot apparatus to obtain reproducible 3-mm-diameter dots in the pattern of a 96-well microtiter plate. Proteins were blotted on the membrane by negative pressure using a vacuum pump. The membrane was dried at 37° for 1 h, blocked in 5% (w/v) fat-free milk powder in PBS (PAA Laboratories), and incubated with the 6E10 antibody (1∶10,000, 4°C over night, Covance). After washing with PBST (PBS containing 0.1% Tween-20), secondary anti-mouse antibody (Promega, Fitchburg, USA) was used at a dilution of 1∶10,000. Blots were developed with ECL enhanced chemiluminescence (Sigma) in an Imager Gel Doc System (Biorad).

### Toxicity tests

To assess proliferation and cell viability, compound-treated cells were subject to MTT and Trypan Blue assays. MTT-Assay (CellTiter 96® Proliferation Assay, Promega) was performed as recommended by provider and absorbance was measured by μQuant spectrophotometer at OD_570_ (Biotek, Winooski, USA). Trypan Blue Stain 0,4% (Invitrogen) was performed as published [32] and percentages of surviving cells were calculated by using a Neubauer counting chamber.

### Western blot

For Western blot analysis of supernatants, 100 µl of media was centrifuged and supernatants were boiled with loading buffer and run on 8% SDS-PAGE, blotted and probed 6E10 (1∶1000, Covance), 3F4 (1∶100, Covance) and rabbit monoclonal β-actin antibody (1∶5000, Sigma), as well as matching secondary antibodies (1∶5000, Promega). For cell lysates procedures were identical but 200 µg of homogenate in RIPA buffer was used. For visualisation and quantification ECL enhanced chemiluminescence (Sigma), Imager Gel Doc System and Quantity One Software (Biorad) was used. For determination of glycosylation ratios between mature and immature APP, band intensities were compared and expressed as ratios.

### Real-time RT-PCR

The RNA was isolated from cell lines using the RNA Miniprep Kit (Stratagen, Basel, Switzerland). The purification of RNA was carried out with RNeasy Lipid Tissue Mini Kit (Qiagen, DE) following the protocol provided by the manufacturer. During purification, samples were treated with RNase-free DNase Set (Qiagen, DE) to avoid later amplification of genomic DNA. The concentration of each sample was obtained from A260 measurements with Nanodrop 1000. RNA integrity was tested using the Agilent 2100 BioAnalyzer (Agilent, US).

### ELISA

For quantification of Aβ_40_ and Aβ_42_ we used Aβ_40_/Aβ_42_-specific sandwich Enzyme Linked-Immuno-Sorbent Assay according to manufacturer's instructions. Briefly, media were centrifuged at 1000 rpm for 5 min (at 4°C), supernatants (50 µl) were added to antibody-coated wells, capture antibody was added and extinction was measured at 450 nm by spectrophotometer (μQuant, BioTek).

### Statistical analysis

In all experiments, means +/− SD are reported. Statistical comparisons among groups were determined using Student's t-test with statistical significance at p-values<0.05 (*), <0.01 (**) and <0.001 (***).

### Identification of similar structures

To assess novelty and to identify similar activities a number of prominent databases were accessed. These included ChEMBL [33], ChemSpider [34], BindingBD [35], PubChem [36], Drug Bank [37], Espacenet [38] and Google Scholar [39] and were accessed between 29^th^ May 2013 and 25^th^ June 2013.

## Results

### A cell-based AD model

**Figure 1:**
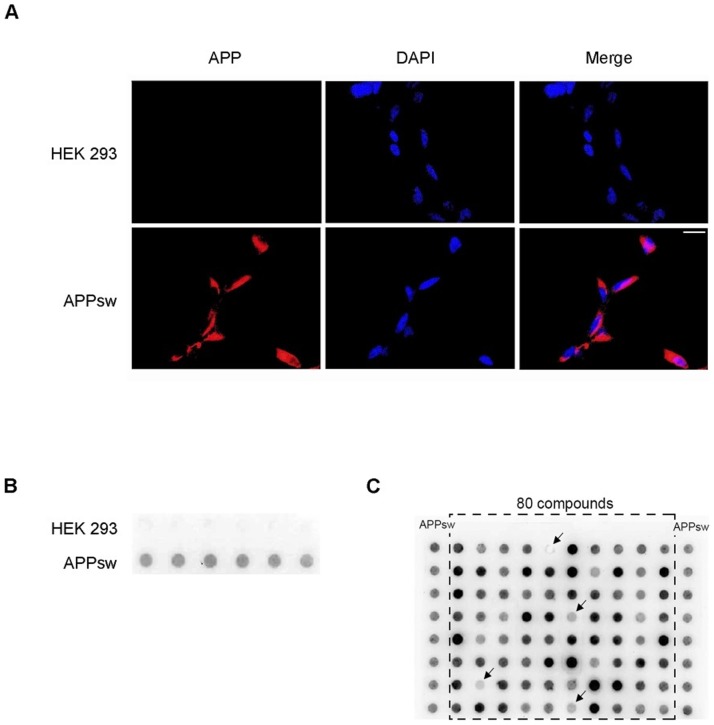
Characterization of a new cell-based assay for screening of APP-lowering small molecules. A) Expression of APP/Aβ in APPsw transfected HEK 293 cells and in HEK 293 cells. APPsw cells and HEK cells were fixed, labelled with 6E10 antibody and stained with Cy3 anti-mouse IgG for detection of APP/Aβ (red). Nuclei were stained with DAPI (blue). Scale bar 25 µm. B) Supernatants of HEK and APPsw cells were characterized via dot blot with the 6E10 antibody. APPsw cells produce a higher level of sαAPP than control HEK cells. C) Representative example of a dot blots from the screening stage of the study. Supernatants of compound-treated APPsw cells and controls (solvent-treated APPsw, extreme left and right lane). With this approach 80 compounds could be assessed in parallel. Only compounds reducing the signal in four independent experiments were evaluated as “positive”.

As a cell culture model for AD we used HEK cells stably transfected with human APP harboring the Swedish mutation (APPsw). Expression of APP and Aβ was assessed by immunohistochemistry for APP/Aβ using the 6E10 antibody. This revealed strong expression of APP/Aβ on plasma membrane as well as intracellularly in nearly 100% of cells (Fig. 1A). Since compounds used for our screen were dissolved in DMSO we excluded unspecific effects of DMSO on expression of APP and generation of Aβ by exposing cells to ascending concentration of DMSO added to the media. There were no significant differences regarding APP expression or generation of Aβ up to a 1/100 dilution of DMSO in media (data not shown).

### Screen for APP-lowering small molecules

The goal of this study was to identify compounds reducing expression of APP. Thus our initial screen using a compound library (ChemBridge DIVERSet™) with 10,000 small molecules designed for high chemical diversity and drug-likeness focused on identifying compounds that decrease the amount of sαAPP in the supernatant.

**Figure 2:**
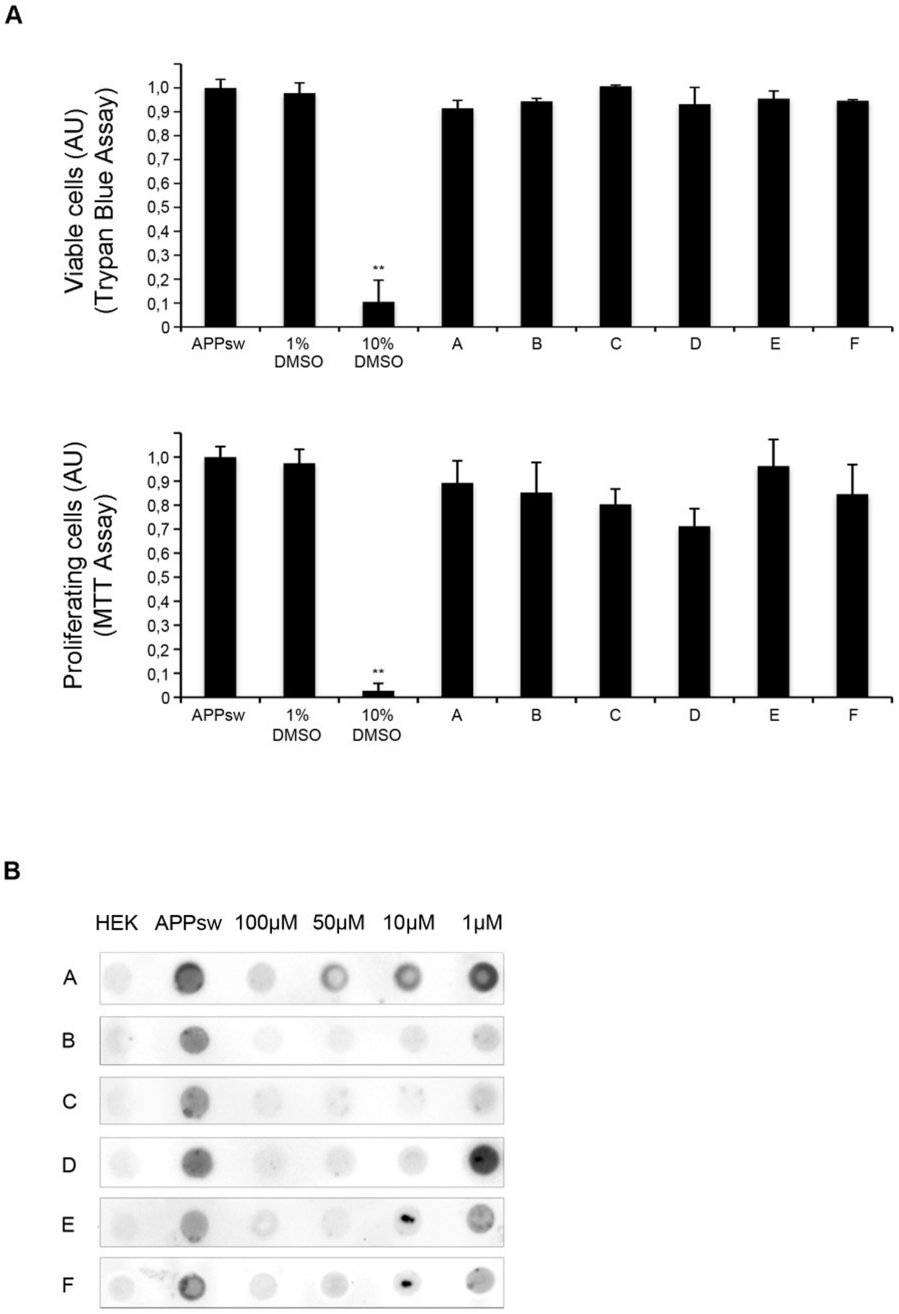
Identification of six compounds lowering APP levels at lower concentrations are non cytotoxic. A) Toxicity assays. Trypan Blue Assay was performed after 3 days of compound incubation. The percentage of surviving cells was calculated. 1%DMSO was used as a negative and 10% DMSO as a positive control. For the MTT assay absorbance of formazan was measured at 570 nm. All experiments were performed in triplets. Results are shown as mean±S.D., n = 3, ***p<0.001. B) The effect of different concentrations was assessed using serial dilutions (100 µM, 50 µM, 10 µM, 1 µM) in four independent experiments. Untransfected HEK 293 cells and solvent-treated APPsw cells were used as controls. A representative example (n = 4) of one blot of the 6 non-cytotoxic compounds is shown.

For this, cells were incubated for 72 hours with above mentioned compounds and supernatants were analyzed by dot blot using the monoclonal antibody 6E10 recognizing sαAPP. The validity of our approach is shown in Fig. 1B where strong uniform signal for sαAPP is only observed in APPsw but not in control HEK cells. For the initial screen, compounds were added at a concentration of 100 µM to single wells in a 96-well format allowing for the simultaneous assessment of 80 compounds (Fig. 1C). Only compounds showing strong sαAPP reduction in four independent experiments were evaluated as “positive”, thus eligible for further investigation. Of 10,000 compounds, 223 were “positive” and were assessed for effectiveness at lower concentrations by incubating cells for 72 hours with compounds at concentrations of 100 µM, 50 µM, 10 µM and 1 µM. As above, supernatants were analyzed by dot blot and compounds were evaluated as “positive” when effects were seen at lower concentrations in four independent experiments. Sixteen compounds were effective at 10 µM and two compounds showed a significant reduction of sαAPP at 1 µM. In order to exclude unspecific effects on cell viability or cellular metabolism, we assessed cytotoxicity of compounds by Trypan Blue assay and influence on proliferation of compounds by MTT-assay. Of the sixteen compounds showing reduction of sαAPP at lower concentrations, six did not show effects on proliferation and were non-cytotoxic (Fig. 2A). Two compounds which decreased sαAPP signal up to 1 µM (Compound B and C) and four compounds that decreased sαAPP signal up to 10 µM (Compound A, D–F) were chosen for further analysis (Fig. 2B). Cytotoxic compounds or compounds with effects only at 100 µM and 50 µM were excluded Fig. S1A, B, C).

**Figure 3:**
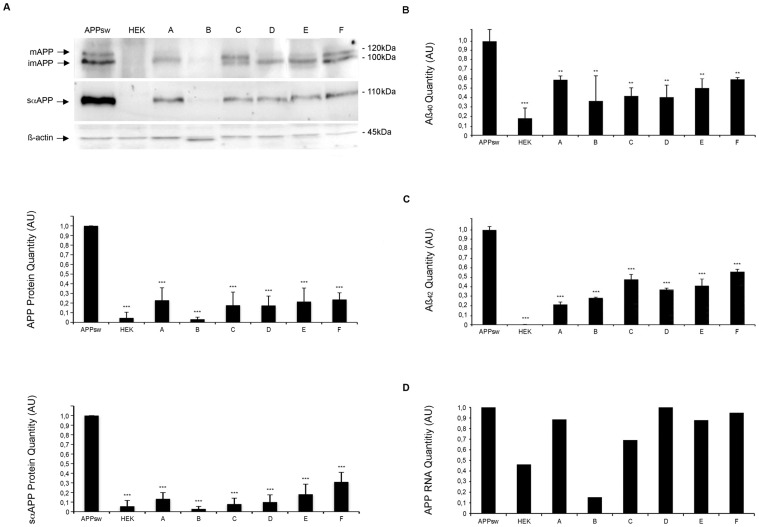
Assessment of APP, Aβ_40_ and Aβ_42_ levels in compounds-treated APPsw cells. A) Western blot of the cell lysates and the supernatants of APPsw cells after 3 day incubation with compounds (10 µM). Arrows indicate fully glycosylated mature, incompletely glycosylated immature APP and sαAPP. β-actin serves as loading control. First Graph showing relative expression of mAPP and imAPP normalized to expression of actin, untreated APPsw controls were set to 1. Results are shown as mean±S.D., n = 5, ***p<0.001. Second Graph showing relative expression of sαAPP normalized to expression of actin, untreated APPsw controls were set to 1. Results are shown as mean±S.D., n = 5, ***p<0.001. B) Aβ_40_ quantification by ELISA with the supernatant of APPsw cells after 3 days of compound incubation (10 µM). Aβ_40_ and Aβ_42_ levels were decreased significantly. Untreated APPsw cells and HEK cells are used as controls. Data are the mean ± S.D., n = 3, **p<0.01, ***p<0.001. C) Aβ_42_ quantification by ELISA with the supernatant of APPsw cells after 3 days of compound incubation (10 µM). Aβ_40_ and Aβ_42_ levels were decreased significantly. Untreated APPsw cells and HEK cells are used as controls. Data are the mean ± S.D., n = 3, **p<0.01, ***p<0.001. D) mRNA levels of APP were measured by RT-PCR in treated APPsw cells and HEK cells, untreated APPsw cells were set to 1. Compound B reduces the amount of APP-mRNA. Data are the mean of two experiments.

Reduction of full-length intracellular/plasma membrane bound APP and sαAPP was confirmed by Western Blot analyses of cell lysates and supernatants 72 hours after incubation with the six selected compounds (at 10 µM). In this analysis, all of the six compounds led to a significant reduction of intracellular/plasma membrane bound APP and sαAPP (Fig. 3A, n = 5, p***<0.001).

To determine if reduction of full-length intracellular/plasma membrane bound APP and sαAPP also applies for neuronal cells, we treated stably transfected neuronal N2asw cells with the six selected compounds (10 µM, 72 hours) and performed Western Blots of cell supernatants and cell lysates. We could not observe cytotoxic effects for these compounds when assessed by MTT-assay (Fig. S2A, n = 3, **p<0.01). Amounts of intracellular/plasma membrane bound APP and *sαAPP* were decreased for all six compounds (Fig. S2B, n = 2).

### Reduction of Aβ_40_ and Aβ_42_ by sαAPP-lowering compounds

To confirm that selected compounds reduce the amount of Aβ_40_ and Aβ_42_ we analyzed supernatants following three-day compound treatment by Aβ_40_ and Aβ_42_ specific ELISA. HEK cells produce small amounts of Aβ_40_, whereas Aβ_42_-production is under the detection limit, whereas APPsw cells produce large amounts of Aβ_40_ and lower amounts of Aβ_42_. All six tested compounds significantly reduced amounts of Aβ_40_ and Aβ_42_ with relative reductions ranging from 36%–59% for Aβ_40_ and 21%–56% for Aβ_42_ (Fig. 3B, C, n = 3, **p<0.01; ***p<0.001).

### Specificity of sαAPP-lowering compounds

To exclude nonspecific reduction of neuronal membrane proteins we assessed expression of PrP^C^ as membrane-bound, highly abundant neuronal protein. PrP^C^ represents a good control protein since it localizes to similar membrane microdomains and its expression is not regulated by APP [40]. With the exception of compound B there was no significant regulation of PrP^C^ by tested compounds (Fig. S3).

### Transcriptional and posttranslational effects of sαAPP-lowering compounds

In order to investigate if decrease of *sαAPP* is due to transcriptional regulation we determined mRNA levels of APP using quantitative RT-PCR using established methods [41]. For compound B we observed transcriptional effects on APP mRNA levels 72 hours after compound treatment whereas for compounds A, C, D, E and F no transcriptional effects were observed (Fig. 3D, n = 2).

APP is N-glycosylated in the ER and cis-Golgi followed by O-glycosylation in medial- and trans-Golgi [42]. The mature form of APP is fully glycosylated (mAPP) whereas the immature form is N-glycosylated (imAPP) [43]. mAPP undergoes cleavage by β- and γ-secretases in the secretory pathway or at the plasma membrane whereas imAPP locates to endoplasmic reticulum or cis-Golgi and is not subject to cleavage [44]. To investigate if this posttranslational modification is changed upon treatment with compounds, we calculated the ratio between mature and immature APP 3 days after of incubation with compounds at 10 µM. All of the tested compounds with the exception of compound A led to a shift of glycosylation pattern towards predominantly immature APP when compared to controls where the ratio between mAPP and imAPP is balanced (Fig. S4, n = 3, *p<0.05).

### Identification of similar structures

**Figure 4:**
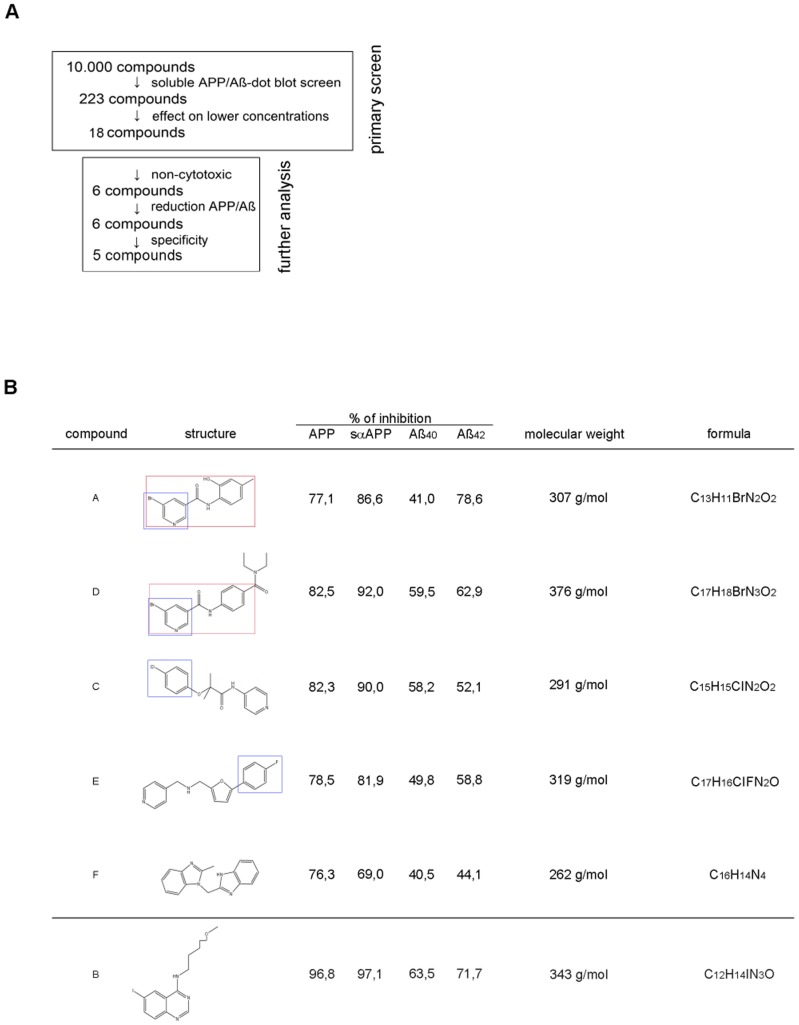
Characterization of the six best compounds. A) Flowchart of the screening of the 10,000 compound library DIVER Set. 10,000 compounds were screened and hits were analyzed by serial dilutions (100 µM, 50 µM, 10 µM, 1 µM). Compounds effective at lower concentrations were checked for cytotoxicity and the non-cytotoxic ones were further analyzed by western blot, ELISA and RT-PCR. B) Structures of the highly potent 5 compounds (A, C–F) in the DIVER Set library which have an specific effect on APP/Aβ-production at a concentration of a minimum of 10 µM and are not cytotoxic. The structure of compound B is added below.

We identified 6 compounds out of the 10,000 which decreased the amount of APP or Aβ at lower concentrations (10 µM) and which were not cytotoxic (Fig. 4A). These findings could be used as starting points for further investigations in developing therapeutic targets. All structures of the 6 compounds are presented in figure 4B. We performed structural comparisons to determine promising structures of the molecules (Fig. S5). The activities and the predicted physicochemical properties of these structures (e.g. cLogP), which are well within the ‘lead-like’ and ‘Drug-like’ space, make them very interesting starting points for Drug Discovery efforts. Two of these six structures, compounds A and D contain a 5-bromonicotinamide moiety. Within the screening set, there were other compounds with this moiety that showed reduced or no activity, indicating some SAR. Thus compounds M and N were weakly active at 100 uM, whilst O and P were inactive at this dose. Compound A has been widely screened within the NIH Molecular Libraries initiative and has only proved active nine times out of the five hundred and ninety one assays in which it has been screened, indicating that it is not a promiscuous structure. None of the assays run are similar to the one of this report. Compound A does appear in a patent containing diverse structures as ‘Ganglioside Biosynthesis Modulators’ [45]. This could be a mode of action by increasing the immature form of APP, even if there is no shift in the mature/immature APP ratio in our Western Blots, but further work would need to be done to confirm this. There is also some SAR around compound C within the 10,000 compounds screened. Compound Q shows weak activity at 100 uM whilst a compound that is very closely related to C, compound R, is inactive. Compound C has also been widely screened within the NIH Molecular Libraries programme and of the 652 assays run, it was only active in 10 – all of these were CYP450 assays. This cytochrome activity is unsurprising for a molecule with an exposed pyridine nitrogen atom and is not likely to be directly related to the activity of this current report. There is also some additional data from this screen for compound B, with compounds S and T both active without toxicity at 50 uM. This structural class has previously been reported to prevent Huntingtin protein aggregation which may have some relevance [46]. However, with the potentially nonspecific effects seen with B, interest in this compound/series is perhaps lower than the others. Few analogues of compound E were assayed, although compound U was inactive at 100 uM suggesting that pyridyl moiety is important for activity. Further investigation in this area of the molecule would also seem sensible, including CYP450 binding. There is again a hint of SAR around compound F - from the small set of related compounds screened, the data suggests that activity may require the benzimidazole and a second basic centre – compound V is inactive at 100 uM whereas compounds W and X show weak activity at this concentration.

## Discussion

We have established a novel cell-based method to screen for compounds lowering APP. Using this approach we have indentified six promising compounds out of 10,000 which significantly reduce levels of APP, Aβ_40_ and Aβ_42_ at lower concentrations, are non-cytotoxic, do not change the metabolic activity and fulfill the terms fur drug-likeness, which is a key fact for developing AD drugs. APP-lowering effects could be seen in non-neuronal HEK cells and in neuronal N2a cells.

There are several possibilities how these compounds interfere with Aβ generation. One possibility is transcriptional downregulation of APP mRNA. APPsw cells mainly express APP 695 which is the APP isoform with the highest propensity to be processed to Aβ [47]. The transcription of APP can be affected by negatively influencing transcription factors or regulatory sequences in promoter regions of APP [48],[49],[50]. Compound B reduces APP mRNA levels and may function on the transcriptional level, although this relative reduction is influenced by stable overexpression of APPsw in our cell culture model. Since this compound also decreased levels of PrP^C^ these actions may be due to unspecific effects making this compound the least attractive compound identified by us.

Another way of interfering with Aβ generation is by influencing the trafficking and proteolytic processing of APP [51],[52]. Once APP reaches the plasma membrane, it is rapidly internalized and subsequently trafficked through endocytic and recycling compartments back to the cell surface or degraded in the lysosome [43]. Disturbed trafficking to the plasma membrane or enhanced degradation in lysosomes, could explain reduced levels of intracellular/plasma membrane APP. A second possibility could be the inhibition of APP maturation by modification of the Golgi apparatus. Recently it could be shown that X11-llike, a neural adaptor protein, regulates intracellular trafficking of APP by this process [44]. O-glycosylation is a prerequisite for γ-secretase cleavage [53], therefore interference with O-glycosylation may result in decreased Aβ production. Although we did not investigate this in detail, for compounds B–F we observed a shift to the immature form of APP.

Finally the sequential cleavage of APP by α-, β- and γ-secretases represents a putative target. Since we designed our compound screen looking at reductions of *sαAPP*, which nicely correlates with APP-levels, direct influence of our compounds on α-, β- and γ-secretases activity is unlikely.

Recently published studies identified lead compounds aimed at treating a wide range of conformational dementias based on their potential to inhibit protein aggregation [54],[31]. Our approach differs from this approach as we screened for compounds aimed at reducing the substrate subject to dementia causing misprocessing. Combining both approaches represents an attractive strategy to indentify highly potent compounds to treat dementia where protein aggregation is causally involved.

In conclusion, we have indentified six compounds which reduce the amount of Aβ_40_ and Aβ_42_ possibly by influencing APP expression (for instance compound B) or APP maturation (compound C, D, E, F). APP-lowering effects could be seen in non-neuronal and neuronal cells.

All compounds with the exception of compound B did not affect expression of other membrane bound neuronal proteins such as PrP^C^. From a Medicinal Chemistry point of view, each of the screening hits constitutes an interesting starting point for Drug Discovery in this vitally important disease area. All of the compounds have excellent potency for lowering APP and predicted analogues may show enhanced profiles.

Future studies will focus on the *in vivo* relevance, optimization of lead structures and on assessing if these compounds have potential for treating other dementias.

## Supporting Information

Figure S1 **Some compounds reducing APP at lower concentrations are cytotoxic.** A) Effect at lower concentrations was assessed using serial dilutions (100 µM, 50 µM, 10 µM, 1 µM) in four independent experiments. Untreated APPsw cells were used as controls. One example of a blot is presented. Compound G, H, J, K, L reduce the APP level in a dose of 50 µM like 33 other compounds. B) I is one of the 10 compounds, which reduce the APP level at 10 µM, but are cytotoxic. Results are shown as mean±S.D., n = 3, ***p<0.001. **C**) Structures of some compounds (G–L) which were cytotoxic or reduce the APPsw level only at a dose of 50 µM.
(TIF)

Figure S2 **Effect of compounds on N2a cells.** **A**) Toxicity assays. For MTT assay absorbance of formazan was measured at 570 nm. All experiments were performed in triplets. 1%DMSO was used as a negative and 10% DMSO as a positive control. We exclude toxic effects on neuronal cells. Results are shown as mean±S.D., n = 3, **p<0.01. B) Western blot of the cell lysates and the supernatants of N2a and N2asw cells after 3 day incubation with compounds (10 µM). Arrows indicate fully glycosylated mature, incompletely glycosylated immature APP and sαAPP. First Graph shows relative expression of mAPP and imAPP normalized to expression of actin, untreated N2asw were set to 1. Results show means of two experiments. Second Graph shows relative expression of sαAPP normalized to expression of actin, untreated N2asw were set to 1. Results show means of two experiments.
(TIF)

Figure S3 **Assessment of specificity.** Western Blot of cells lysates and supernatants of APPsw cells after 3 day incubation with compounds (10 µM). Arrow indicates PrP^C^ (diglycosylated, monoglycosylated and unglycosylated). β-actin serves as a marker for equal loading. Histogram showing relative expression of PrP^C^ normalized to expression of actin, untreated APPsw controls were set to 1. Results are shown as mean±S.D., n = 3, *p<0.05.
(TIF)

Figure S4 Effect of compounds on APP-glycosylation. Analysis of Glycosylation ratio. The ratio of mature to immature APP was calculated. Compound B, C, D, E and F lead to a shift to immature APP. Results are shown as mean±S.D., n = 3, *p<0.05.
(TIF)

Figure S5 **Structural comparison.** We checked similarity to other compounds of the library (compound M–X) and their appearance in other databases to determine promising structures of the molecules.
(TIF)
