# Supplementary figures and images for: Amyloid-Precursor-Protein-Lowering Small Molecules for Disease Modifying Therapy of Alzheimer's Disease
Source: PLoS One. 2013 Dec 18;8(12):e82255. doi: 10.1371/journal.pone.0082255 (PMC3867334; doi:10.1371/journal.pone.0082255)

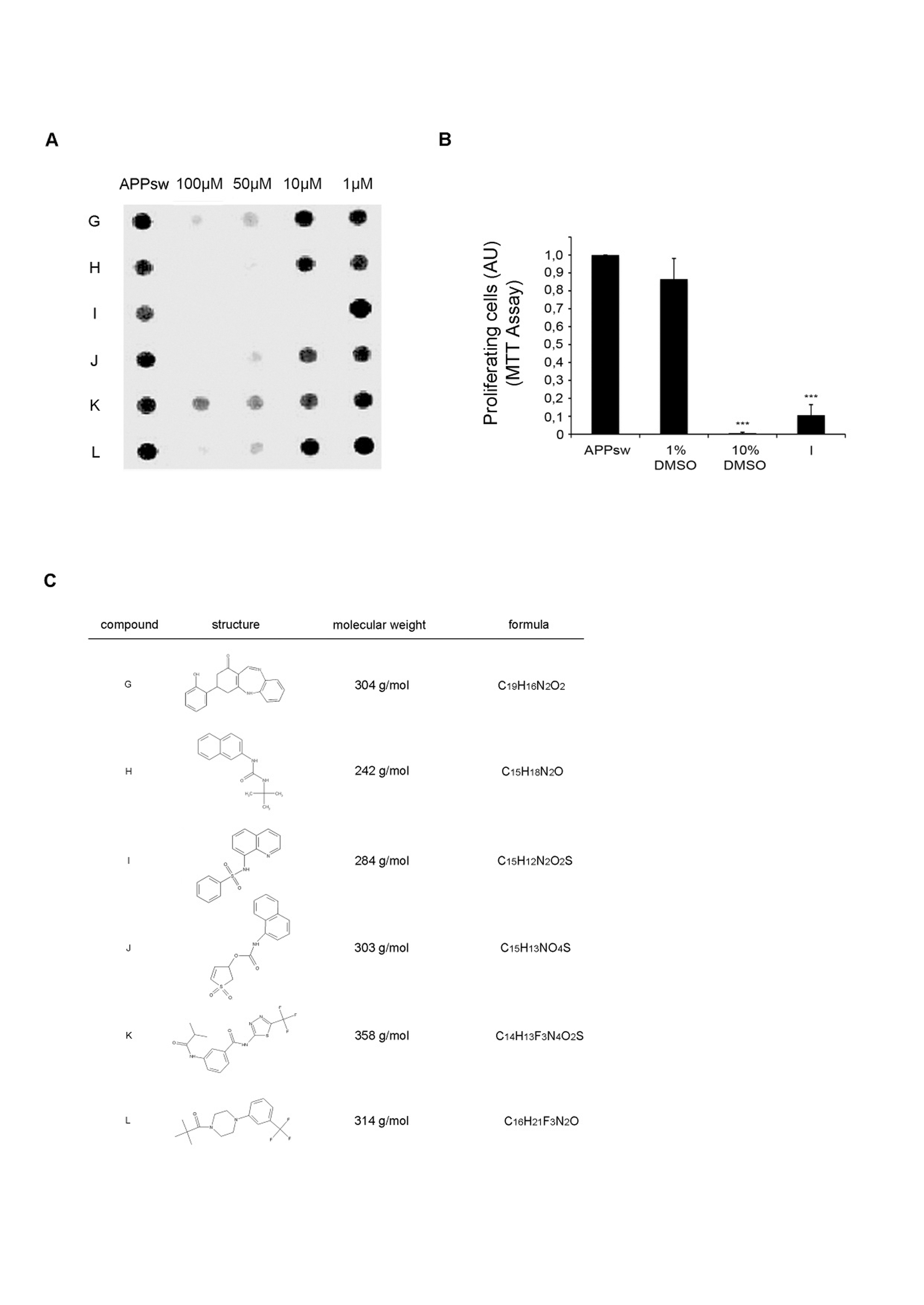

Supplement: Figure S1 — Some compounds reducing APP at lower concentrations are cytotoxic. A) Effect at lower concentrations was assessed using serial dilutions (100 µM, 50 µM, 10 µM, 1 µM) in four independent experiments. Untreated APPsw cells were used as controls. One example of a blot is presented. Compound G, H, J, K, L reduce the APP level in a dose of 50 µM like 33 other compounds. B) I is one of the 10 compounds, which reduce the APP level at 10 µM, but are cytotoxic. Results are shown as mean±S.D., n = 3, ***p<0.001. C) Structures of some compounds (G–L) which were cytotoxic or reduce the APPsw level only at a dose of 50 µM. (TIF) [file pone.0082255.s001.tif]

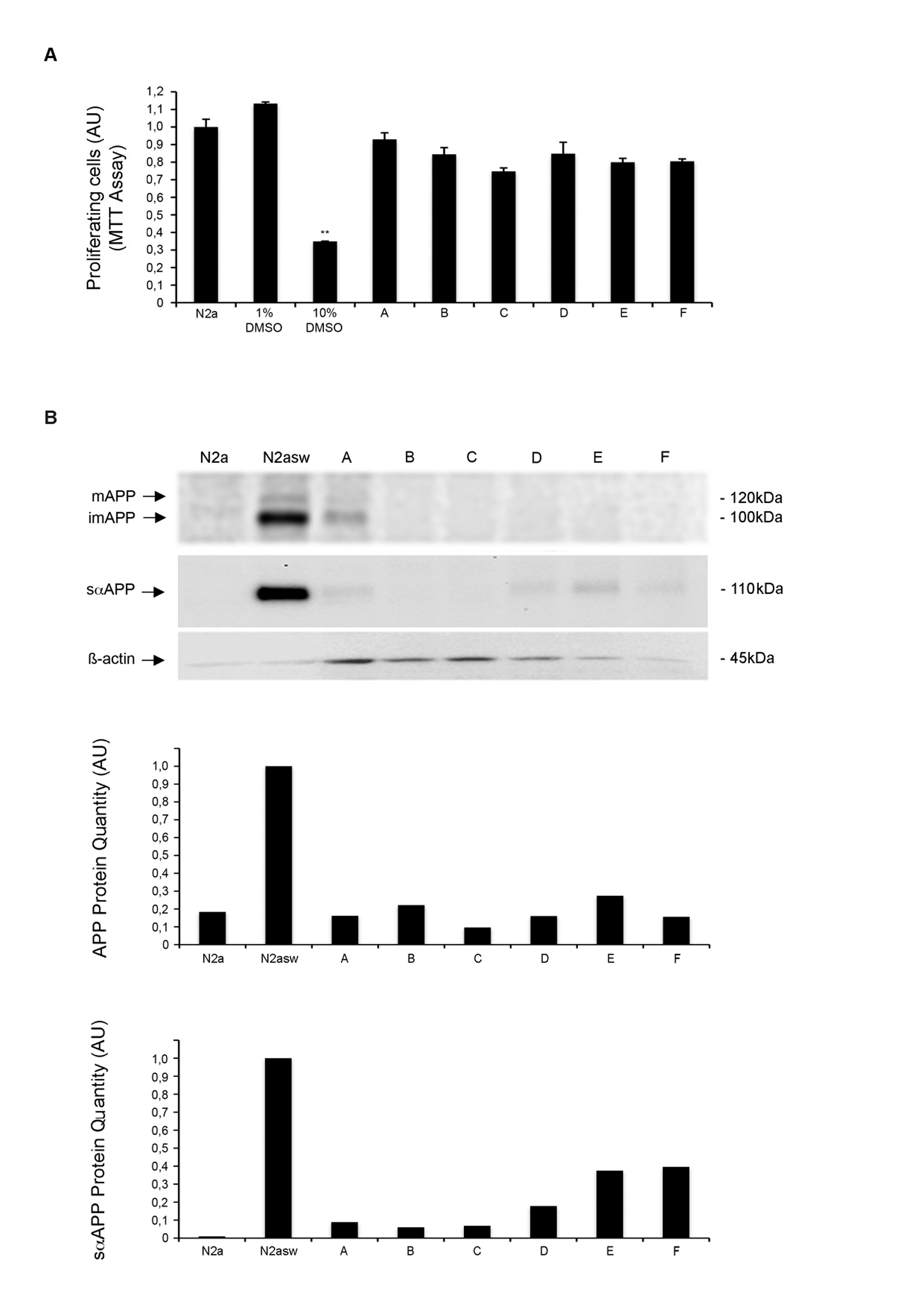

Supplement: Figure S2 — Effect of compounds on N2a cells. A) Toxicity assays. For MTT assay absorbance of formazan was measured at 570 nm. All experiments were performed in triplets. 1%DMSO was used as a negative and 10% DMSO as a positive control. We exclude toxic effects on neuronal cells. Results are shown as mean±S.D., n = 3, **p<0.01. B) Western blot of the cell lysates and the supernatants of N2a and N2asw cells after 3 day incubation with compounds (10 µM). Arrows indicate fully glycosylated mature, incompletely glycosylated immature APP and sαAPP. First Graph shows relative expression of mAPP and imAPP normalized to expression of actin, untreated N2asw were set to 1. Results show means of two experiments. Second Graph shows relative expression of sαAPP normalized to expression of actin, untreated N2asw were set to 1. Results show means of two experiments. (TIF) [file pone.0082255.s002.tif]

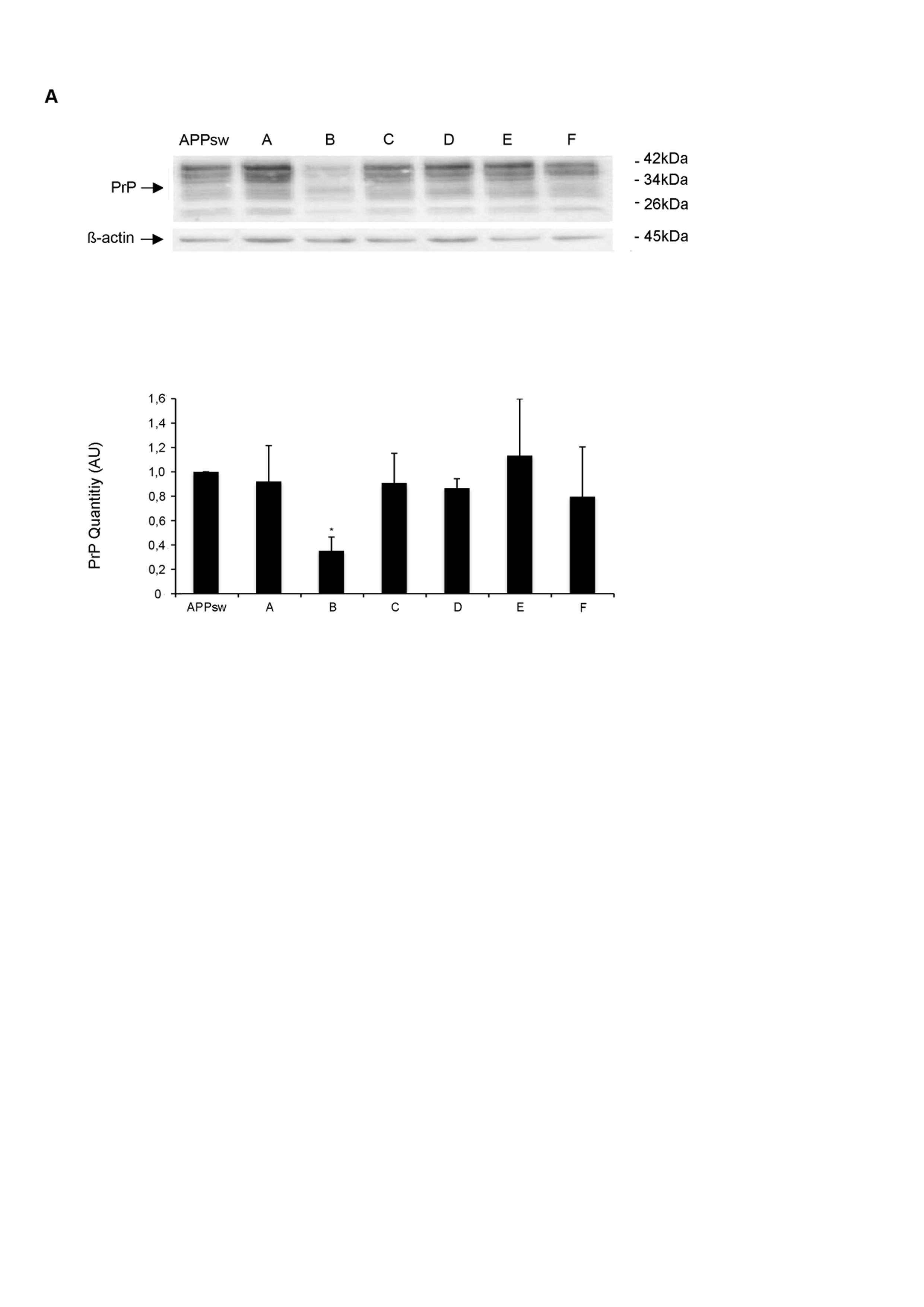

Supplement: Figure S3 — Assessment of specificity. Western Blot of cells lysates and supernatants of APPsw cells after 3 day incubation with compounds (10 µM). Arrow indicates PrPC (diglycosylated, monoglycosylated and unglycosylated). β-actin serves as a marker for equal loading. Histogram showing relative expression of PrPC normalized to expression of actin, untreated APPsw controls were set to 1. Results are shown as mean±S.D., n = 3, *p<0.05. (TIF) [file pone.0082255.s003.tif]

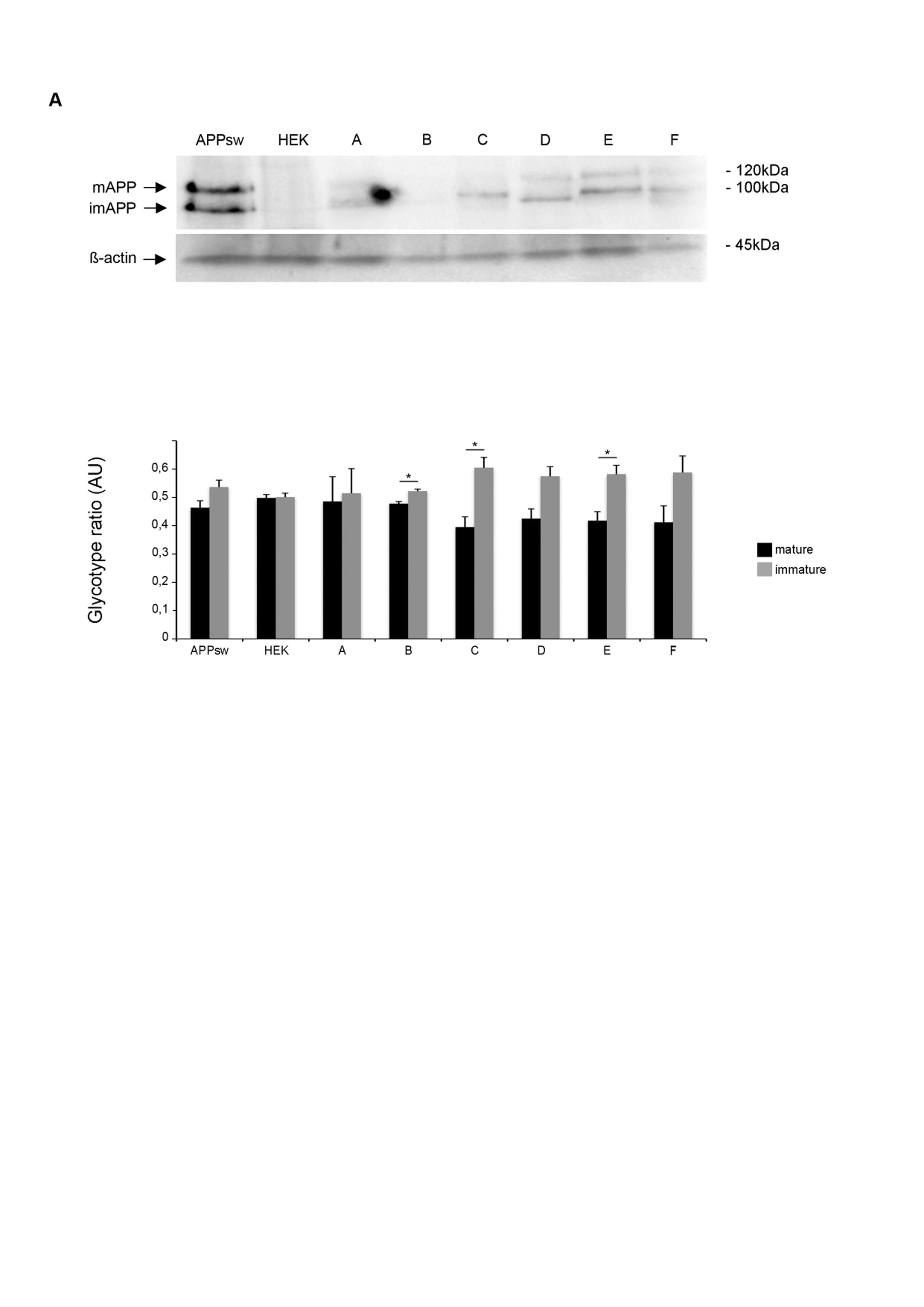

Supplement: Figure S4 — Effect of compounds on APP-glycosylation. Analysis of Glycosylation ratio. The ratio of mature to immature APP was calculated. Compound B, C, D, E and F lead to a shift to immature APP. Results are shown as mean±S.D., n = 3, *p<0.05. (TIF) [file pone.0082255.s004.tif]

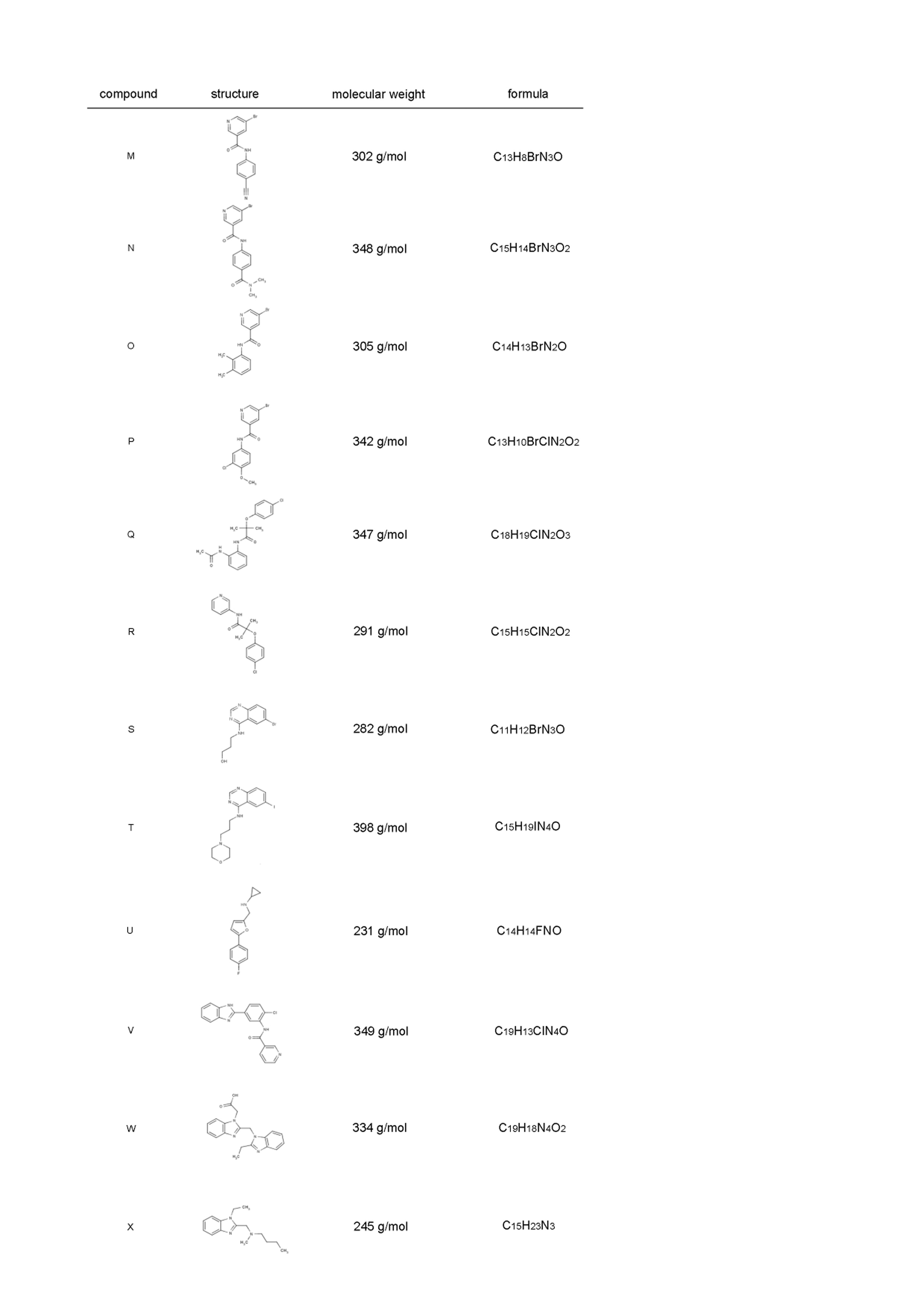

Supplement: Figure S5 — Structural comparison. We checked similarity to other compounds of the library (compound M–X) and their appearance in other databases to determine promising structures of the molecules. (TIF) [file pone.0082255.s005.tif]
